# Dual regulation of p53 by the ribosome maturation factor SBDS

**DOI:** 10.1038/s41419-020-2393-4

**Published:** 2020-03-20

**Authors:** Qian Hao, Jieqiong Wang, Yajie Chen, Shanshan Wang, Mingming Cao, Hua Lu, Xiang Zhou

**Affiliations:** 1 Fudan University Shanghai Cancer Center and Institutes of Biomedical Sciences, Fudan University, Shanghai, 200032 China; 2 Department of Oncology, Shanghai Medical College, Fudan University, Shanghai, 200032 China; 3 Department of Biochemistry & Molecular Biology and Tulane Cancer Center, Tulane University School of Medicine, New Orleans, LA 70112 USA; 4 The Shanghai Key Laboratory of Medical Epigenetics and the International Co-laboratory of Medical Epigenetics and Metabolism, Institutes of Biomedical Sciences, Fudan University, Shanghai, 200032 China; 5 Key Laboratory of Breast Cancer in Shanghai, Fudan University Shanghai Cancer Center, Fudan University, Shanghai, 200032 China; 6 Present Address: Department of Biochemistry & Molecular Biology, Tulane University School of Medicine, New Orleans, LA 70112 USA

**Keywords:** Tumour-suppressor proteins, Stress signalling

## Abstract

The Shwachman-Bodian Diamond syndrome (SBDS)-associated gene, *SBDS*, is involved in rRNA synthesis and ribosome maturation, but the role of SBDS in cancer is largely elusive. In this study, we found that SBDS is often overexpressed or amplified in human cancers, and high level of endogenous SBDS is significantly associated with unfavorable prognosis. Conversely, knockdown of SBDS leads to p53 stabilization and activation through the ribosomal stress-RPL5/RPL11-MDM2 pathway, resulting in the repression of cancer cell proliferation and invasion. Interestingly, ectopic SBDS in the nucleoplasm also suppresses tumor cell growth and proliferation in vitro and in vivo. Mechanistically, ectopically expressed SBDS triggered by, for example, ribosomal stress binds to the transactivation domain of p53 and perturbs the MDM2–p53 interaction, consequently leading to impaired p53 ubiquitination and proteasomal degradation. Altogether, our finding for the first time demonstrates the dual functions of SBDS in cancer development by coordinating ribosome biogenesis and p53 activity.

## Introduction

*SBDS* was designated because its mutation is highly associated with Shwachman-Diamond syndrome (SDS) or Shwachman-Bodian Diamond syndrome (SBDS) characterized with pancreatic dysfunction, hematologic failure, skeletal abnormalities, and neurological phenotypes^1,2^. SDS is also considered as a type of ribosomopathies, as the SBDS protein is involved in rRNA processing and assembly of the mature 80 S ribosome^3^. Several genetic models have been generated to elucidate the essential role of SBDS during embryonic development^4^. Conditional knockout of SBDS in the distinct murine osteoprogenitors was found to result in disordered hematopoiesis or bone homeostasis, respectively^5,6^. The mice with pancreatic-specific depletion of SBDS manifested the SDS-associated pancreatic insufficiency^7^. This is probably because ablation of SBDS in the murine pancreas induced p53 activation^8^, similar to the mechanism for other ribosomophathies^3^. However, the zebrafish models with SBDS depletion presented p53-independent SDS phenotypes^9,10^, as simultaneous silencing of p53 failed to rescue those defects^10^. Thus, it remains unclear and tempting to explore whether and how SBDS regulates p53 activity, particularly, during the development of cancer.

The tumor suppressor p53 prevents malignancies by maintaining genomic stability, triggering cell death, inhibiting epithelial–mesenchymal transition (EMT) and metastasis, and intervening cancer metabolism^11,12^. The E3-ubiquitin ligase MDM2, encoded by a p53 target gene, is the core repressor of p53 by mediating its proteasomal degradation, translational inhibition, and functional inactivation^13^. The MDM2–p53 circuit is subjected to multiple regulations in response to different stress signals or in the context of different cancers^14,15^. Recently, a dozen of ribosomal proteins (RPs) have been found to be dissociated from the pre-ribosomes and interact with MDM2 leading to p53 stabilization and activation upon ribosomal stress^16,17^. These findings lead to the development of several anticancer strategies by activating the tumor-suppressive function of these RPs in the wild-type p53-sustaining tumors ^17,18^.

In the present study, we found that upregulation of SBDS is associated with unfavorable prognosis in a broad spectrum of human cancers. Conversely, ablation of endogenous SBDS prohibits cancer cell proliferation and invasion through the RPL5/RPL11-MDM2–p53 signaling pathway. In contrast to the natively expressed SBDS that acts as an oncogenic protein, aberrant expression of SBDS in the nucleoplasm in response to ribosomal stress suppresses tumor cell growth in vitro and in vivo by inhibiting MDM2-mediated p53 degradation. Collectively, our study unveils a dual regulator, SBDS, of the MDM2–p53 circuit and suggests that SBDS could be a prognostic biomarker and molecular target for cancer treatment.

## Materials and methods

### Plasmids and antibodies

The Flag-tagged pEnter-SBDS plasmid was purchased from Vigene Biosciences (Shandong, China). The Myc-tagged SBDS was generated by inserting the full-length cDNA amplified by PCR from pEnter-SBDS into the pcDNA/Myc-His vector, using the following primers, 5’-CCGCTCGAGATGTCGATCTTCACCCC-3’ and 5’-CGCGGATCCTTCAAATTTCTCATCTCCTTC-3’. The plasmids encoding HA-MDM2, p53, Flag-p53 fragments, His-Ub were described previously^19^. The lentivirus-based SBDS-expressing plasmid or shRNAs were constructed using the vectors pLenti-EF1a-EGFP-P2A-Puro-CMV-3Flag and pLKD-CMV-G&PR-U6, respectively (OBio Technology, Shanghai, China). The shRNA targeting sequences were obtained from Sigma-Aldrich and as follows, 5’-GCCAACAGTTAGAAATCGTAT-3’ and 5’-GCCAAATACTTGCTTAAACTA-3’. The anti-Flag (Cat. No. F1804, Sigma-Aldrich, St louis, MO, USA), anti-Myc (Cat. No. 60003-1, Proteintech, Wuhan, Hubei, China), anti-HA (Cat. No. 2367, Cell Signaling Technology, Danvers, MA, USA), anti-SBDS/mouse (Cat. No. sc-271350, D-9, Santa Cruz Biotechnology, Santa Cruz, CA, USA), anti-SBDS/rabbit (Cat. No. ab154222, Abcam, Cambridge, MA, USA), anti-p53/mouse (Cat. No. sc-126, DO-1, Santa Cruz Biotechnology), anti-p53/rabbit (Cat. No. ab179477, Abcam), anti-MDM2 (Cat. No. ab16895, 2A10, Abcam), anti-GAPDH (Cat. No. 60004-1-Ig, Proteintech), anti-β-actin (Cat. No. ARG62346, Proteintech), anti-RPL5 (Cat. No. ab86863, Abcam), anti-RPL11 (Cat. No. ab79352, Abcam), anti-p21 (Cat. No. 2947, Cell Signaling Technology), anti-PUMA (Cat. No. 12450, Cell Signaling Technology), and anti-fibrillarin (Cat. No. 16021-1-AP, Proteintech) were commercially purchased.

### Cell culture and transient transfection

Human cancer cell lines H460 and H1299 were purchased from American Type Culture Collection. HCT116^p53+/+^ and HCT116^p53−/−^ were generous gifts from Dr. Bert Vogelstein at the John Hopkins Medical institutes. SK-MEL-147 was a generous gift from Dr. Shaomeng Wang at University of Michigan, Ann Arbor. Cells were cultured in Dulbecco’s modified Eagle’s medium supplemented with 10% fetal bovine serum, 50 U/ml penicillin and 0.1 mg/ml streptomycin, and maintained at 37 °C in a 5% CO_2_ humidified atmosphere. All the cell lines were mycoplasma-free and authenticated by PCR analysis. Cells seeded on the plate overnight were transfected with plasmids or siRNA as indicated in figure legends using Hieff Trans Liposomal transfection reagent following the manufacturer’s protocol (Yeasen, Shanghai, China). Cells were harvested at 30–72 h post transfection for future experiments. The cycloheximide (CHX) and proteasome inhibitor MG132 were purchased from Sigma-Aldrich.

### Reverse transcription and quantitative real-time PCR

Total RNA was isolated from cells using RNAiso Plus (Takara, Dalian, Liaoning, China) following the manufacturer’s protocol. Total RNAs of 0.5 to 1 µg were used as templates for the reverse transcription using PrimeScript RT Reagent Kit with gDNA Eraser (Takara). Quantitative PCR (qPCR) was conducted using TB Green Premix Ex Taq (Tli RNaseH Plus) according to the manufacturer’s protocol (Takara). The primers for human BTG2, BAX, MDM2, p21, PUMA, and GAPDH were previously described ^19^.

### Immunoblotting

Cells were harvested and lysed in lysis buffer consisting of 50 mM Tris/HCl (pH7.5), 0.5% Nonidet P-40 (NP-40), 1 mM EDTA, 150 mM NaCl, 1 mM dithiothreitol (DTT), 0.2 mM phenylmethylsulfonyl fluoride, 10 µM pepstatin A and 1 µg/ml leupeptin. Equal amounts of clear cell lysate (20-80 µg) were used for immunoblotting (IB) analysis as described previously ^20^.

### In vivo ubiquitination assay

H1299 cells were transfected with plasmids encoding p53, HA-MDM2, His-Ub or Flag-SBDS as indicated in the figure legends and treated with MG132 for 4–6 hr before being harvested. At 48 h after transfection, cells were harvested and split into two aliquots, one for IB and the other for the ubiquitination assay. In brief, cell pellets were lysed in buffer I (8 M urea, 0.1 M Na_2_HPO_4_/NaH_2_PO_4_ (pH 8.0), 10 mM Tris-HCl (pH 8.0), 10 mM β-mercaptoethanol, 5 mM Imidazole) and incubated with Ni-NTA beads (Takara) that capture His-tagged proteins/complex at room temperature for 4 h. Beads were washed twice with buffer I, then twice with buffer II (8 M urea, 0.1 M Na_2_HPO_4_/NaH_2_PO_4_ (pH 6.3), 10 mM Tris-HCl (pH 6.3), 10 mM β-mercaptoethanol). The captured proteins were eluted and analyzed by IB with the indicated antibodies in the figure legends.

### Immunoprecipitation

Immunoprecipitation (IP) was conducted using antibodies as indicated in the figure legends. In brief, 500–1000 µg of proteins were incubated with the indicated antibody at 4 °C for 4 hr or overnight. Protein A or G beads (Santa Cruz Biotechnology) were then added and the mixture was left to incubate at 4 °C for additional 1–2 hr. The beads were washed at least three times with lysis buffer. Bound proteins were detected by IB with antibodies as indicated in the figure legends.

### Proximity ligation assay

The proximity ligation assay (PLA) was performed to detect protein–protein interactions in cells using the Duolink In Situ Red Starter Kit (DUO92101, Sigma-Aldrich) according to the manufacturer’s instruction. In brief, cells were fixed with methanol in − 20 °C for overnight and blocked with 8% goat serum for 1 hr, followed by incubation of primary antibodies (anti-SBDS/mouse, 1:2000 dilution; anti-p53/rabbit, 1:1500 dilution; anti-MDM2/mouse, 1:1500 dilution) in 3% goat serum in 4 °C for overnight. Cells were then sequentially incubated with PLA probes in 37 °C for 1 hr, ligase solution in 37 °C for 30 min, and amplification-polymerase solution in 37 °C for 100 min. At last, the cell slides were mounted by Duolink In Situ Mounting Medium with 4′,6-diamidino-2-phenylindole (DAPI) and analyzed by a confocal microscope (Leica SP5, Wetzlar, Germany).

### Immunofluorescence staining and confocal microscopy

Cells were fixed with methanol in −20 °C for overnight. The fixed cells were washed by phosphate-buffered saline (PBS) and blocked with 8% bovine serum albumin (BSA) in PBS for 1 hr followed by incubation with primary antibodies (anti-SBDS, 1:200 dilution; anti-p53, 1:200 dilution) in 2% BSA in 4 °C for overnight. The cells were then washed and incubated with the corresponding secondary antibodies and DAPI. Images were acquired with a confocal microscope (Leica SP5, Wetzlar, Germany).

### RNA interference

The siRNAs against SBDS, RPL5, or RPL11^21^ were commercially purchased (GenePharma, Shanghai, China). An amount of 40–100 nM of siRNAs was introduced into cells using Hieff Trans Liposomal transfection reagent following the manufacturer’s protocol. Cells were harvested 48–72 hr after transfection for IB or qRT-PCR. The siRNA sequences used were as follows, siSBDS: CGAAAUCGCCUGCUACAAA, siRPL5: GGAGGAGAUGUAUAAGAAA, and siRPL11: GGAACUUCGCAUCCGCAAA.

### Generating stable cell lines

Lentivirus plasmids encoding SBDS or the empty vector were packaged and purified as described (OBio Technology, Shanghai, China). HCT116^p53+/+^ and HCT116^p53−/−^ cells were infected with appropriate amount of viruses for overnight and then the medium was changed. The stable cells were selected with 1 µg/ml puromycin.

### Flow cytometry analysis

Cells transfected with siRNAs as indicated in the figure legends were fixed with ethanol overnight and stained in 500 µl of propidium iodide (Sigma-Aldrich) stain buffer (50 µg/ml PI, 200 µg/ml RNase A, 0.1% Triton X-100 in phosphate-buffered saline) at 37 °C for 30 min. The cells were then analyzed for DNA content and sub-G1 distribution using a FC500 MPL flow cytometer (Beckham coulter, Indianapolis, IN, USA). The FITC Annexin V Apoptosis Detection Kit I (BD Biosciences, San Diego, CA, USA) was used for apoptosis analysis according to the manufacturer’s instruction. In brief, cells were washed twice with cold PBS, resuspended in binding buffer, and stained with FITC Annexin V and PI for 15 min at RT.

### Cell viability assay

The Cell Counting Kit-8 (CCK-8) (Dojindo Molecular Technologies, Japan) was used according to the manufacturer’s instructions. Cells of 2000–5000 were seeded per well in 96-well culture plates at 12 hr post transfection. Cell viability was determined by adding WST-8 at a final concentration of 10% to each well, and the absorbance of the samples was measured at 450 nm using a Microplate Reader every 24 hr for 4–5 days.

### Colony formation assay

Cells were trypsinized and seeded with the same amount on six-well plates 12–18 hr after siRNA or plasmid transfection. The medium was changed every 3 days until the colonies were visible. Puromycin was added in the medium to select the positive cells with plasmid expression. The visible colonies were then fixed by methanol and stained by crystal violet solution at RT for 30 min. ImageJ was used for quantification of the area of colonies.

### Transwell invasion assay

The assay was performed using the Transwell chamber inserts in a 24-well plate. In all, 5 × 10^4^ cells suspended in 100 µL of serum-free medium were added to the upper chamber. The lower chambers were filled with the normal culture medium. After culture for 36–48 h at 37 °C, the cells on the upper surface were scraped and washed away, and the cells on the lower surface were fixed with methanol and stained with 0.1% crystal violet. The number of invaded cells was counted in at least three randomly selected fields under an optical microscope by image J software.

### Mouse xenograft study

Seven-week-old female BALB/c nude mice were purchased from Shanghai SLAC Laboratory Animal Co.,Ltd. The sample size was estimated based on the need for statistical power. Mice were randomized into four groups (eight mice in each) and subcutaneously inoculated with 1 × 10^7^ HCT116^p53+/+^ or HCT116^p53−/−^ cells stably expressing plasmid encoding SBDS or the empty vector. The investigator was blinded to the group allocation when the inoculation was conducted. Tumor growth was monitored with electronic digital calipers in two dimensions. Tumor volume was calculated with the formula: tumor volume (mm^3^) = (length × width^2^)/2. Mice were killed by euthanasia and tumors were harvested for future analyses. The animal protocols were in compliance with ethical regulations and approved by the Animal Welfare Committee of Shanghai Medical College at Fudan University. To confirm p53 activation by SBDS in vivo, the tumors were disrupted and lysed in the RIPA buffer (50 mM Tris-HCl (pH 8.0), 5 mM EDTA, 1% NP-40, 0.5% Deoxycholate, 0.1% SDS, 150 mM NaCl) and subjected to qRT-PCR and IB analyses.

### Data of cancer patients

Genetic alterations of SBDS and RP genes were analyzed using the TCGA database (www.cbioportal.org)^22,23^. The expression of SBDS in cancer versus normal tissues was analyzed using the Oncomine database (www.oncomine.org). For survival analysis of colorectal adenocarcinoma patients, the RNA-Seq and clinical data were retrieved from TCGA and subjected to the Gehan–Breslow–Wilcoxon analysis. Cancer patient survival was also analyzed by the Kaplan–Meier method using the KM plotter database (kmplot.com/analysis/)^24^. The colorectal cancer tissue microarray service was provided by Shanghai Outdo Biotech Co., Ltd. Informed consent was obtained from all patients, and the study was approved by the ethics committee of Fudan University Shanghai Cancer Center.

### Statistics

All in vitro experiments were performed in biological triplicate. The Student’s *t* test or one way analysis of variance was performed to evaluate the differences between two groups or more than two groups. The variance between the groups that are being statistically compared is similar. The Kaplan–Meier statistics were used to analyze the significant difference of patient survival. The Cox univariate proportional hazards regression models was used to determine the independent clinical factors based on the investigated variables. *p* < 0.05 was considered statistically significant, and asterisks represent significance in the following way: *p < 0.05; **p < 0.01. Quantitative data are presented as mean ± SD.

## Results

### SBDS is upregulated in human cancers and associated with unfavorable prognosis

**Fig. 1:**
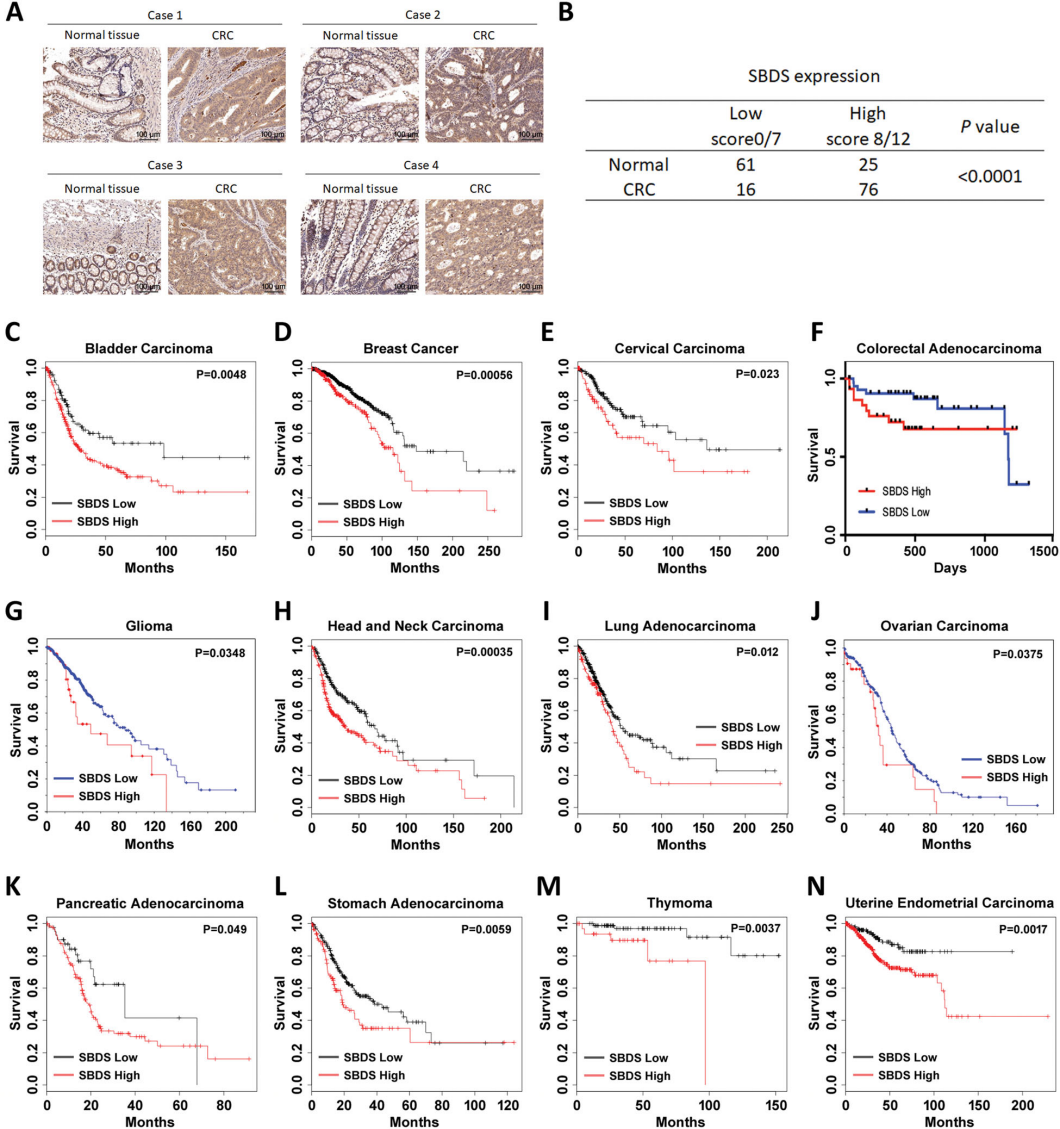
High level of SBDS is associated with unfavorable cancer prognosis. **a**, **b** Colorectal cancer tissue array analysis shows that SBDS is significantly overexpressed in cancer versus normal tissues. **c**–**n** The Kaplan–Meier survival analysis indicate that high expression of endogenous SBDS predicts poor patient survival in a wide spectrum of human cancers, including bladder cancer **c**, breast cancer **d**, cervical cancer **e**, colorectal cancer **f**, glioma **g**, head and neck cancer **h**, lung cancer **i**, ovarian cancer **j**, pancreatic cancer **k**, stomach cancer **l**, thymoma **m**, and uterine endometrial cancer **n**.

Ribosomophathies are frequently accompanied by increased predisposition for cancers, such as acute myeloid leukemia, lymphoma, osteosarcoma, head and neck cancer, and others^3^. Indeed, several RP-encoding genes associated with Diamond-Blackfan anemia or 5q-syndrome were found to be engaged in tumorigenesis by regulating p53, TAp73, c-MYC, or NF-κB signaling pathway^16,17^. It remains unclear if and how the SDS-associated gene SBDS has a role in cancer development. By mining the Oncomine database, we found that SBDS is upregulated in multiple human cancers compared with the normal tissues (Fig. S1A). In addition, the TCGA database showed that SBDS is preferentially upregulated or amplified in all types of cancers examined (Fig. S1B). We also performed colorectal cancer tissue array analysis and found that SBDS is significantly overexpressed in colorectal cancer versus adjacent normal tissues (Fig. 1a, b). More importantly, the Kaplan–Meier analysis revealed that increased expression of SBDS is significantly associated with unfavorable prognosis of a wide range of human solid tumors (Fig. 1c–n). These findings suggest for the first time that SBDS may act as an oncogenic protein by driving cancer progression, and thus we decided to further investigate the role of SBDS in the development of solid malignancies.

### Ablation of endogenous SBDS activates p53 pathway

**Fig. 2:**
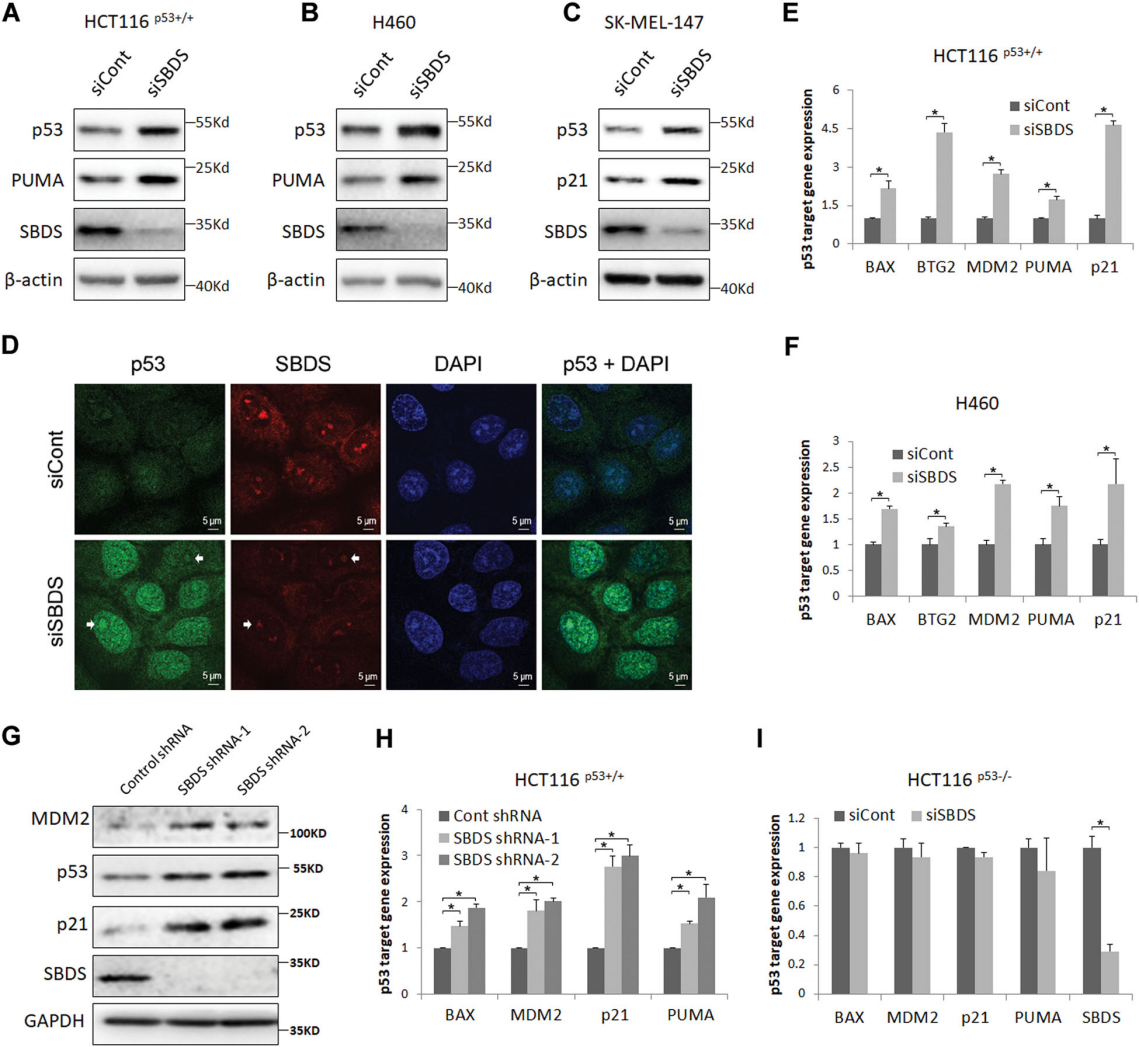
Ablation of endogenous SBDS activates p53 pathway. **a**–**c** Knockdown of SBDS elevates the protein levels of p53 and its target genes, PUMA and p21. HCT116^p53+/+^ **a**, H460 **b**, and SK-MEL-147 **c** cell lines were transfected with control or SBDS siRNA followed by IB analyses using antibodies as indicated. **d** Knockdown of SBDS induces p53 accumulation in the nucleus. Cells were transfected with control or SBDS siRNA followed by IF staining assay. The white arrows indicate the nucleoli. **e**, **f** Knockdown of SBDS in HCT116^p53+/+^ **e** and H460 **f** cells elevates the mRNA expression of p53 target genes, including BAX, BTG2, MDM2, PUMA, and p21, by qRT-PCR analyses. **g** Knockdown of SBDS by lentivirus-delivered shRNAs elevates the protein expression of p53 and its target genes, MDM2 and p21. HCT116^p53+/+^ cells were infected with lentiviruses and harvested for IB analysis using antibodies as indicated. **h** Knockdown of SBDS in HCT116^p53+/+^ cells by lentivirus-delivered shRNAs elevates the mRNA expression of p53 target genes, including BAX, MDM2, PUMA, and p21, by qRT-PCR analyses. **i** Knockdown of SBDS in HCT116^p53−/−^ cells does not affect the expression of p53 target genes at RNA levels. The same experiment was conducted using p53-deficient HCT116 cells as that done for **e**.

It was shown by our group and others that impairment of the 40 S or 60 S ribosome subunit by depleting RPS-proteins (e.g., RPS14 and RPS19) or RPL-proteins (e.g., RPL23, RPL29, and RPL30) leads to ribosomal stress and consequent p53 activation^20,25–27^. We inquired if ablation of endogenous SBDS can do so as well, because SBDS is responsible for ribosome biogenesis^28,29^. First, we performed siRNA-mediated silencing of SBDS in several wild-type p53-sustaining cancer cell lines, including colorectal cancer cell line HCT116^p53+/+^, lung cancer cell line H460 and melanoma cell line SK-MEL-147 (Fig. 2a–c). As expected, knockdown of SBDS elevated the protein levels of p53 and its target genes, PUMA and p21 (Fig. 2a–c). Also, an immunofluorescence (IF) staining assay was conducted to validate the accumulation of p53 in the nucleus upon SBDS depletion (Fig. 2d). We also observed that knockdown of SBDS leads to the increase of p53 in the nucleoli with SBDS itself as a nucleolar marker (Fig. 2d), which is consistent with previous studies showing that blocking the proteasome promotes nucleolar localization of p53^30,31^. To test if this depletion boosts p53’s transcriptional activity, we examined the mRNA expression of more p53 target genes by qRT-PCR. As shown in Fig. 2e, f, knockdown of SBDS resulted in the upregulation of multiple p53 target genes, including BAX, BTG2, MDM2, PUMA, and p21, at the mRNA level in HCT116^p53+/+^ and H460 cell lines. To exclude the possible off-target effect of the siRNA, we exploited two more shRNAs by targeting different sites of SBDS. Consistently, knockdown of SBDS by each shRNA markedly induced p53 and its target gene expression in HCT116^p53+/+^ cells (Fig. 2g, h). In addition, ablation of SBDS in HCT116^p53−/−^ cells does not affect the expression of p53 target genes (Fig. 2i). Therefore, these results indicate that deprivation of SBDS leads to activation of the p53 signaling pathway in various cancer cells that harbor wild-type p53.

### Ablation of endogenous SBDS induces RPL5- and RPL11-mediated p53 stabilization

**Fig. 3:**
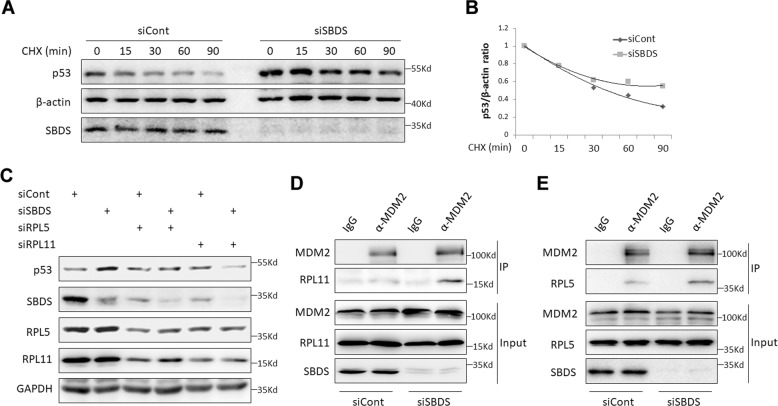
Ablation of endogenous SBDS induces RPL5- and RPL11-dependent p53 stabilization. **a**, **b** Knockdown of SBDS prolongs p53 protein half-life. HCT116^p53+/+^ cells were transfected with control or SBDS siRNA for 48–72 hr. CHX was supplemented into the medium for the indicated time before the cells were harvested for IB analysis **a**. Quantification of the p53/β-actin ratios is shown in the **b**. **c** RPL5 and RPL11 are required for SBDS depletion-induced p53 activation. HCT116^p53+/+^ cells were transfected with the indicated siRNAs for 48–72 hr and harvested for IB analysis using the antibodies as indicated. **d** Knockdown of SBDS enhances the RPL11-MDM2 interaction. HCT116^p53+/+^ cells were transfected with control or SBDS siRNA for ~48 hr. The proteasome inhibitor MG132 was supplemented into the medium for 4 hr before the cells were harvested for co-IP-IB assays using antibodies as indicated. **e** Knockdown of SBDS enhances the RPL5-MDM2 interaction. The same experiments were conducted as described in **d**, except that the anti-RPL5 antibody was used.

Next, we wanted to determine whether ablation of SBDS, like several RPs^20,25,32^, may cause p53 protein stabilization. We performed the CHX-chase experiment and found that SBDS depletion indeed leads to p53 stabilization as indicated by its extended protein half-life (Fig. 3a, b). It is known that RPL5 and RPL11 are critical for ribosomal stress-mediated p53 activation by interacting with MDM2 in the nucleoplasm^33^. Thus, we tested if RPL5 and RPL11 are required for p53 stabilization induced by SBDS depletion. As shown in Fig. 3c, knockdown of RPL5 or RPL11 abrogated SBDS depletion-caused p53 induction. We also noticed that knockdown of RPL5 diminishes RPL11 expression and vice versa, which is in line with a previous study showing that RPL5 and RPL11 can mutually stabilize each other^34^. Furthermore, we found that SBDS depletion increases the interactions of RPL11 and RPL5 with MDM2 (Fig. 3d, e). Altogether, these results demonstrate that SBDS depletion can cause ribosomal stress that triggers the interaction of RPL5/RPL11 with MDM2, consequently leading to p53 stabilization and activation.

### Endogenous SBDS is required for cancer cell propagation partially dependent of p53

**Fig. 4:**
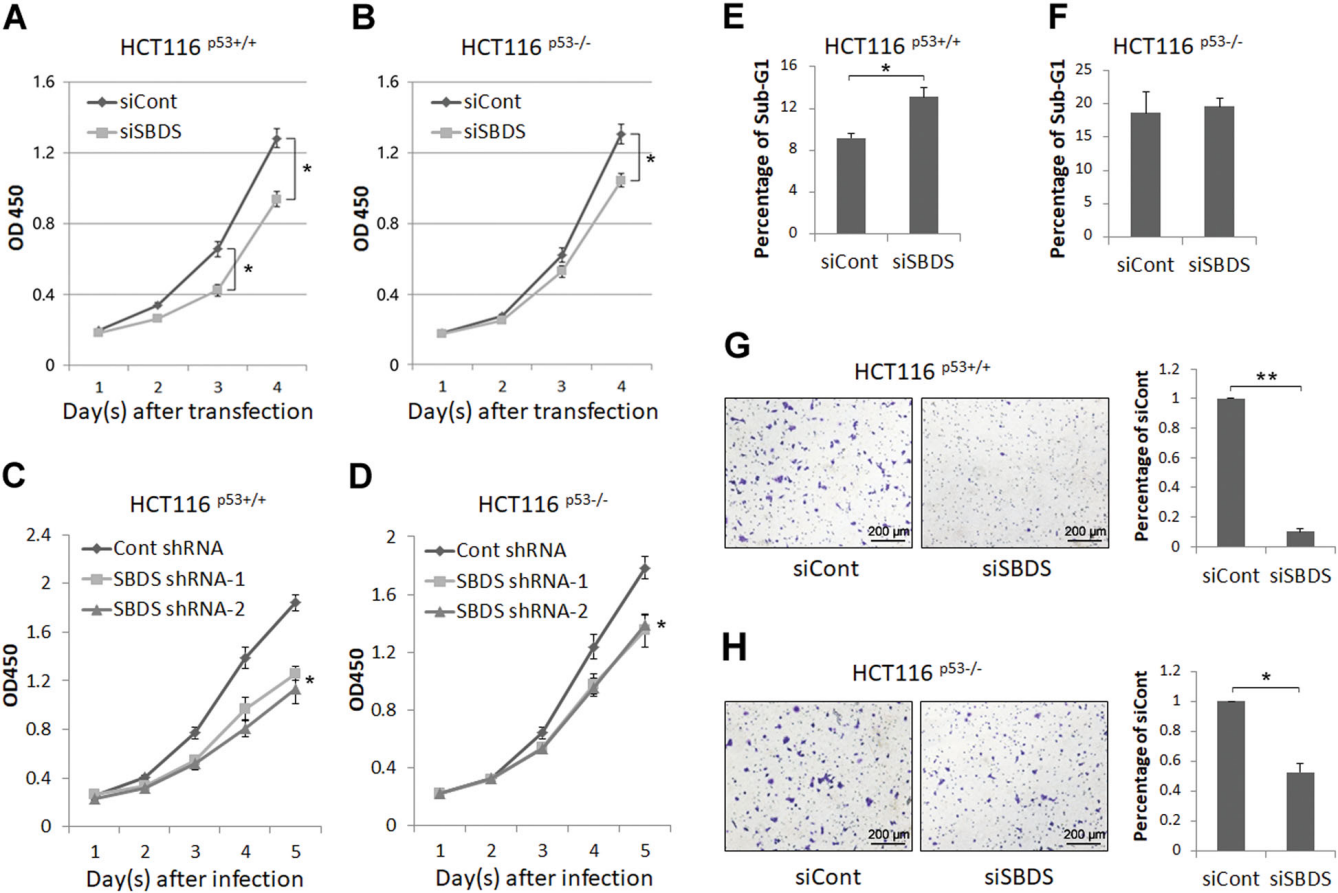
Ablation of endogenous SBDS inhibits cancer cell growth and invasion. **a**, **b** Knockdown of SBDS by siRNA inhibits proliferation of HCT116^p53+/+^ cells more dramatically than that of HCT116^p53−/−^ cells. Cells were transfected with control or SBDS siRNA and re-seeded in 96-well plate followed by the cell viability assay. **c**, **d** Knockdown of SBDS by shRNAs inhibits proliferation of HCT116^p53+/+^ cells more dramatically than that of HCT116^p53−/−^ cells. Cells were infected with control or SBDS-shRNA lentiviruses and re-seeded in 96-well plate followed by the cell viability assay. **e**, **f** Knockdown of SBDS induces sub-G1 accumulation of HCT116^p53+/+^ cells but not HCT116^p53−/−^ cells. Cells were transfected with control or SBDS siRNA for 48–72 hr followed by flow cytometry analyses. **g**, **h** Knockdown of SBDS prevents invasion of HCT116^p53+/+^ cells more dramatically than that of HCT116^p53−/−^ cells. Cells were transfected with control or SBDS siRNA followed by the transwell cell invasion assay.

As high expression of SBDS was associated with poor prognosis (Fig. 1) and its depletion-induced p53 activation (Figs. 2, 3), we inquired if endogenous SBDS is essential for cancer cell growth and survival. To test this idea, we performed a cell viability assay and found that SBDS ablation significantly reduces HCT116^p53+/+^ cell proliferation from day 3 after transfection (Fig. 4a), whereas exerts a moderate inhibitory effect on HCT116^p53−/−^ cell proliferation on day 4 after transfection (Fig. 4b). Consistently, ablation of SBDS by shRNAs more drastically induced tumor cell growth arrest in HCT116^p53+/+^ cells than that in HCT116^p53−/−^ cells (Fig. 4c, d). These effects were not owing to the variation of gene knockdown efficiency, as SBDS expression was dramatically dampened by siRNA or shRNAs to a comparable extent in both cell lines (Fig. S2). In addition, we conducted flow cytometry analysis and found that SBDS depletion leads to augmented sub-G1 accumulation of HCT116^p53+/+^ cells, whereas this depletion barely affects apoptosis of HCT116^p53−/−^ cells (Fig. 4e, f). As p53 was shown to suppress EMT and metastasis by, for example, transcriptionally inducing the expression of miR-200c^35^, we also examined if SBDS is involved in cancer cell invasion by regulating p53 activity. The results of the transwell invasion assay revealed that loss of SBDS markedly repressed the mobility of HCT116^p53+/+^ cells compared with that of HCT116^p53−/−^ cells (Fig. 4g, h). Moreover, we evaluated the long-term cell growth by performing the colony formation assay. Interestingly, these two isogenic cell lines displayed similar colony-forming ability by knocking down SBDS (Fig. S3). This result along with a previous study^8^ suggests that persistent depletion of SBDS may ultimately lead to translation insufficiency and growth inhibition, regardless of p53 expression, in cancer cells. Taken together, these findings demonstrate that ablation of endogenous SBDS suppresses cancer cell proliferation and invasion partially through the RP-MDM2–p53 signaling pathway, but its long-term deficiency is more detrimental to cell survival owing to its essential role in ribosome biogenesis.

### Ectopic expression of SBDS enhances p53 stabilization and activation

**Fig. 5:**
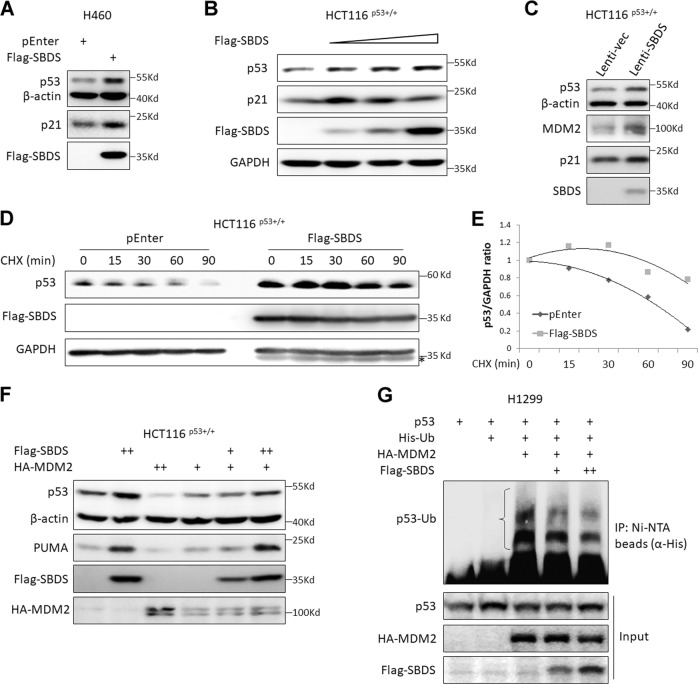
Ectopic SBDS induces p53 stabilization and activation. **a** Ectopic expression of SBDS induces the expression of p53 and its target gene p21 in H460 cells. Cells were transfected with the empty vector or SBDS plasmid followed by IB analysis. **b** Ectopic SBDS induces the expression of p53 and its target gene p21 in a dose-dependent manner in HCT116^p53+/+^ cells. Cells were transfected with increased dosage of SBDS plasmid and followed by IB analysis. **c** Lentivirus-delivered ectopic SBDS induces the expression of p53 and its target genes, MDM2 and p21. HCT116^p53+/+^ cells were infected with control or SBDS-expressing lentiviruses followed by IB analysis. **d**, **e** Ectopic SBDS prolongs p53 protein half-life. HCT116^p53+/+^ cells were transfected with the empty vector or SBDS plasmid for 30–48 hr and treated with CHX for the indicated time followed by IB analysis. The ratios of p53 to GAPDH were presented in **e**. **f** Ectopic SBDS counteracts MDM2-induced p53 degradation. HCT116^p53+/+^ cells were transfected with combinations of Flag-SBDS, HA-MDM2, and empty vector followed by IB analysis. **g** Ectopic SBDS impairs MDM2-induced p53 ubiquitination. H1299 cells were transfected with combinations of p53, His-Ub, HA-MDM2, and Flag-SBDS followed by in vivo ubiquitination assay and IB analysis. Poly-ubiquitination of p53 was indicated by the open curly brace.

As our data as presented above showed that robust expression of endogenous SBDS is required for p53 homeostasis, as SBDS depletion provokes p53 activation (Figs. 2, 3), we wondered if ectopic SBDS regulates p53 activity or not. To our surprise, ectopic expression of SBDS in H460 cells elevated the expression of p53 and p21 (Fig. 5a). Consistently, ectopic SBDS activated p53 in a dose-dependent manner in HCT116^p53+/+^ cells (Fig. 5b). Also, lentivirus-directed expression of SBDS markedly induced p53 and its target gene expression (Fig. 5c). The IF staining result revealed that overexpression of SBDS leads to the elevation of p53 in the nucleus as well as the nucleolus (Fig. S4). These data suggested that ectopic SBDS might stabilize p53. Indeed, ectopic SBDS extended p53 protein half-life as shown in our CHX-chase analysis (Fig. 5d, e). As the E3 ligase MDM2 is the master regulator of p53, we tested if ectopic SBDS can counteract MDM2-mediated p53 degradation. As shown in Fig. 5f, overexpression of MDM2 reduced p53 protein level as expected (lanes 3 and 4 vs. lane 1), whereas simultaneous overexpression of SBDS neutralized the inhibitory effect of MDM2 on p53 in a dose-dependent manner (lanes 5 and 6 vs. lane 4), suggesting that ectopic SBDS stabilize p53 by subverting the MDM2-E3 activity toward p53. Consistently, ectopic expression of SBDS exerted a dose-dependent effect in inhibiting p53 ubiquitination by MDM2 (Fig. 5g). Therefore, these results demonstrate that ectopic SBDS suppresses MDM2-mediated p53 ubiquitination and consequent degradation.

### SBDS perturbs MDM2–p53 interaction by competing for binding to the TA domain of p53 in response to ribosomal stress

**Fig. 6:**
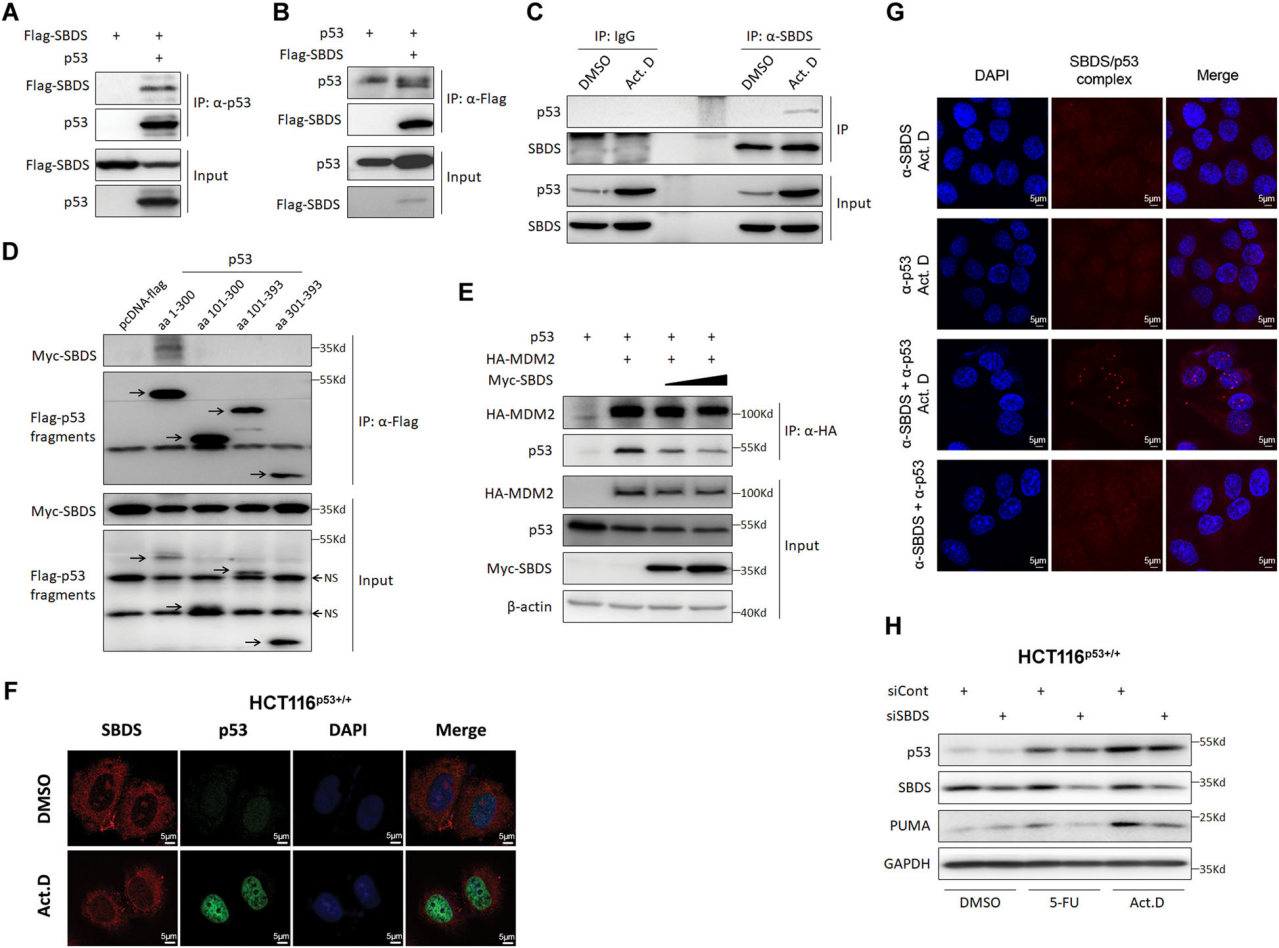
Ectopic SBDS disrupts MDM2–p53 interaction by binding to p53-TA domain in response to ribosomal stress. **a**, **b** Ectopic SBDS binds to exogenous p53. H1299 cells were transfected with the indicated plasmids followed by co-IP assays using α-p53 **(a)** or α-Flag **(b)**. The bound complexes were subjected to IB analyses. **c** SBDS binds to p53 in response to ribosomal stress. HCT116^p53+/+^ cells were treated with or without Act. D followed by co-IP-IB analyses using antibodies as indicated. **d** Ectopic SBDS binds to the TA domain of p53. Cells were transfected with the indicated plasmids followed by co-IP-IB analyses. The arrows indicate different p53 fragments. **e** Ectopic SBDS disrupts MDM2–p53 interaction. Cells were transfected combinations of the indicated plasmids followed by co-IP-IB analyses. **f** Ribosomal stress induces ectopic expression of SBDS in the nucleoplasm. Cells were treated with or without Act. D followed by IF staining using antibodies as indicated. **g** SBDS binds to p53 in the nucleus in response to ribosomal stress. Cells were treated with or without Act. D followed by the PLA assay using antibodies as indicated. **h** SBDS is required for ribosomal stress-triggered p53 activation. Cells were transfected with control or SBDS siRNA for 48 to 72 hr and treated with 5-FU or Act. D before harvest for IB analysis.

Next, we sought to elucidate the mechanism underlying the inhibition of MDM2 activity toward p53 by SBDS. To this end, we first examined if SBDS binds to MDM2 by performing co-IP-IB assays. As illustrated in Fig. S5, we only detected residual binding of SBDS to MDM2 compared with the RPS14–MDM2 interaction in the same assay^20^, suggesting that SBDS might not act like MDM2-binding RPs, such as RPS14, to inactivate MDM2 activity toward p53. We then tested if it binds to p53 or not. Interestingly, we found that ectopic SBDS interacts with exogenous p53 by reciprocal co-IP-IB assays (Fig. 6a, b). To confirm this interaction, we examined if endogenous SBDS and p53 can bind to each other or not. Remarkably, endogenous SBDS bound to endogenous p53 in cells that were treated with Actinomycin D (Act. D), although their interaction was hardly detectable in unstressed cells (Fig. 6c), suggesting that the SBDS-p53 binding is responsive to ribosomal stress. By mapping the SBDS-binding domain(s) of p53, we further showed that SBDS interacts with the N-terminal transactivation (TA) domain (amino acids 1–100), but not the amino acids 101–300, 101–393, or 301–393, of the p53 protein (Fig. 6d). It is known that the TA domain is also required for the binding of MDM2^36^, and thus we reasoned that SBDS may interfere with MDM2–p53 interaction by binding to the same region. Indeed, ectopic expression of SBDS markedly disturbs the MDM2–p53 interaction in a dose-dependent manner (Fig. 6e).

As endogenous SBDS bound to endogenous p53 only in response to ribosomal stress caused by Act. D treatment (Fig. 6c), we then wondered if ribosomal stress could alter the cellular localization of SBDS, allowing its interaction with p53 in the nucleoplasm. SBDS was mainly detected in the nucleolus and cytoplasm, whereas p53, though at quite a low level, was only detected in the nucleoplasm under the non-stress condition (Fig. 6f). This observation could explain why endogenous SBDS hardly binds to p53 under the normal culture condition (Fig. 6c). Interestingly, some SBDS molecules were detected and colocalized with highly expressed p53 in the nucleoplasm when cells were treated with Act. D (Fig. 6f), suggesting that SBDS could bind to and activate p53 in the nucleoplasm in response to ribosomal stress caused by this drug. To test this, we conducted a PLA. As a positive control, the MDM2/p53 complex was first probed and shown in the nuclei of cells treated with Act. D (Fig. S6), indicating the reliability of PLA for detection of protein interactions in situ. As illustrated in Fig. 6g, the SBDS/p53 complexes were clearly detected as fluorescent spots in the nucleus upon ribosomal stress. These signals were very specific because the cells incubated with a single primary antibody or those without Act. D treatment did not show any visible PLA signals. In line with these results, endogenous SBDS was required for p53 activation by ribosomal stress, as knockdown of SBDS reduced the 5-FU- or Act. D-induced expression of p53 and PUMA (Fig. 6h). Collectively, these results explicitly demonstrate that SBDS interacts with p53 in the nucleus and consequently induces its activity and downstream signaling pathway in response to ribosomal stress, and suggest that SBDS might play a tumor-suppressive role that will be addressed as follows.

### SBDS suppresses cancer cell proliferation in vitro and tumor growth in vivo

**Fig. 7:**
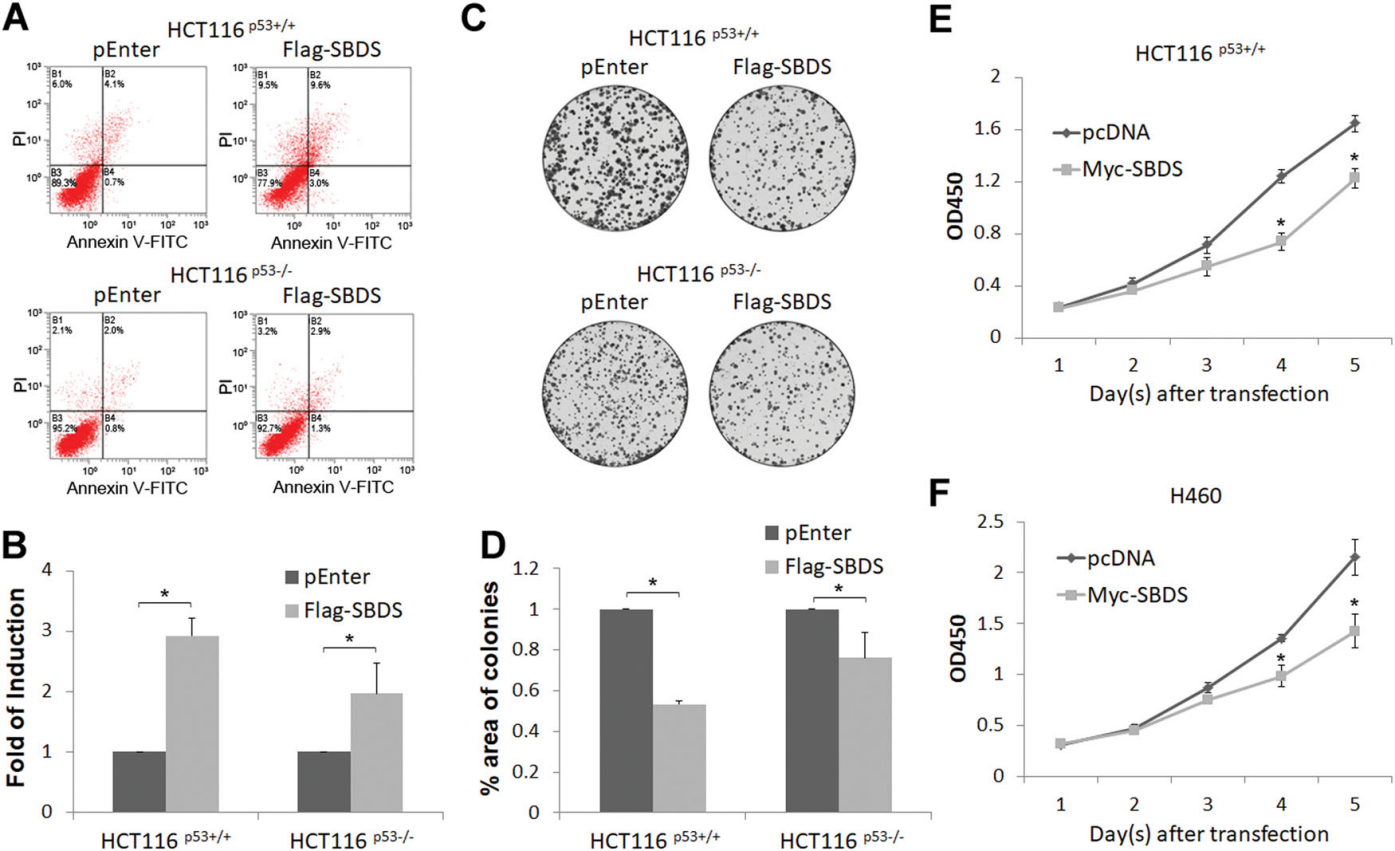
Ectopic SBDS suppresses cancer cell growth in vitro. **a**, **b** SBDS promotes apoptosis more dramatically in HCT116^p53+/+^ cells than that in HCT116^p53−/−^ cells. Cells were transfected with control or SBDS plasmid followed by the Annexin V-FITC and flow cytometry analysis. Quantification of the apoptotic cells are shown in **b**. **c**, **d** SBDS more dramatically suppresses colony-forming ability of HCT116^p53+/+^ cells than that of HCT116^p53−/−^ cells. Cells were transfected with control or SBDS plasmid followed by the colony formation assay. **e**, **f** SBDS inhibits proliferation of HCT116^p53+/+^ **e** and H460 cells **f**. Cells were transfected with control or SBDS plasmid followed by the cell viability assay.

To determine whether SBDS has a tumor-suppressive role, we first performed flow cytometry analysis of colon cancer cells after overexpression of SBDS. As shown in Fig. 7a, b, ectopic SBDS markedly promoted apoptosis of HCT116^p53+/+^ cells, though it also induced apoptosis of HCT116^p53−/−^ cells to a less extent, suggesting that SBDS can at least in part induce p53-dependent apoptosis. Consistently, as revealed by our colony formation assays, ectopic SBDS repressed colony-forming ability of HCT116^p53+/+^ cells more dramatically than that of HCT116^p53−/−^ cells (Fig. 7c, d). Also, ectopic SBDS inhibited proliferation of HCT116^p53+/+^ and H460, both of which contained wild-type p53, as shown by cell viability assays (Fig. 7e, f). Hence, these results indicate that ectopic SBDS induces apoptosis and inhibits cell proliferation and colony formation partially dependently of p53.

**Fig. 8:**
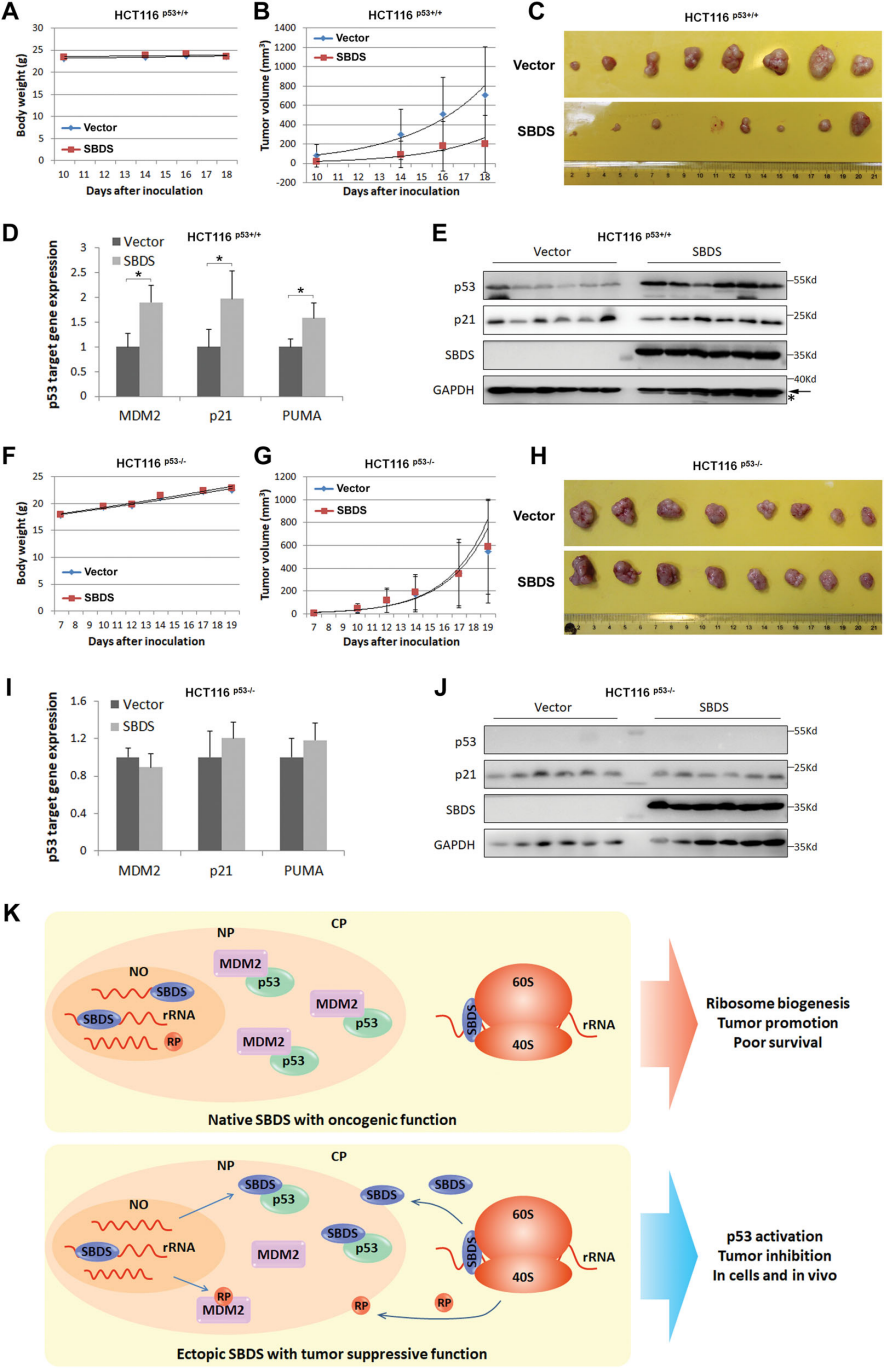
Ectopic SBDS suppresses tumor growth in vivo. **a** Ectopic SBDS does not affect body weights of the mice bearing tumors derived from HCT116^p53+/+^ cells. **b** Ectopic SBDS suppresses growth of the xenograft tumors derived from HCT116^p53+/+^ cells in vivo. **c** Representation of the dissected tumors derived from HCT116^p53+/+^ cells expressing the empty vector or ectopic SBDS. **d**, **e** Ectopic SBDS activates p53 pathway in tumors derived from HCT116^p53+/+^ cells. The tumors were homogenized and lysed for qRT-PCR **d** or IB analysis **e**. **f** Ectopic SBDS does not affect body weights of the mice bearing tumors derived from HCT116^p53−/−^ cells. **g** Ectopic SBDS does not affect growth of the xenograft tumors derived from HCT116^p53−/−^ cells in vivo. **h** Representation of the dissected tumors derived from HCT116^p53−/−^ cells expressing the empty vector or ectopic SBDS. **i**, **j** Ectopic SBDS does not affect p53 target gene expression in tumors derived from HCT116^p53−/−^ cells. The tumors were homogenized and lysed for qRT-PCR **i** or IB analysis **j**. **k** Working model of dual regulation of p53 activity by SBDS. Under the normal culture condition, SBDS mostly reside in the nucleolus and cytoplasm to conduct oncogenic function by boosting ribosome biogenesis and maintaining p53 at a low expression level (upper panel). In response to ribosomal stress, SBDS translocates to the nucleoplasm to execute a tumor-suppressive role by interacting with p53 and dissociating the MDM2–p53 complex (lower panel).

To further expand the biological significance of the above cell-based findings, we examined if ectopic SBDS suppresses tumor growth in xenograft mouse models. HCT116^p53+/+^ and HCT116^p53−/−^ cells stably overexpressing empty vector or SBDS were generated by lentivirus-mediated transduction and then used in the following xenograft experiments. The ectopic SBDS-expressed HCT116^p53+/+^ cells and the control cells were inoculated subcutaneously into the BALB/c nude mice, and the body weights and tumor volumes were measured at Day 10, 14, 16, and 18 after inoculation. In line with the cell-based results, the xenograft tumor growth was significantly inhibited by ectopic expression of SBDS as compared to the control group (Fig. 8b), whereas the body weights were not markedly affected (Fig. 8a). Also, the tumors derived from the ectopic SBDS-expressed HCT116^p53+/+^ cells are markedly smaller in size than those from the control cells (Fig. 8c). Furthermore, ectopic SBDS induced p53 activity in vivo, as both mRNA and protein levels of p53 target genes were elevated in the ectopic SBDS-expressed tumors (Fig. 8d, e), which is again consistent with the cell-based results (Fig. 5). To determine whether the in vivo tumor-suppressive activity of ectopic SBDS is dependent on p53 status, we also generated the xenograft model using HCT116^p53−/−^ cells. As shown in Fig. 8f–j, ectopic SBDS did not influence the mouse body weights (Fig. 8f), xenograft tumor volumes or sizes (Fig. 8g, h) dramatically. Consistently, the expression of p53 target genes was not affected by ectopic SBDS in these p53-negative tumors (Fig. 8i, j). Taken together, our results substantiate the concept that SBDS can execute p53-dependent tumor-suppressive functions in cells and in vivo.

## Discussion

Ribosome biogenesis is finely tuned with cell growth and proliferation. Malignant tumor cells harbor more active nucleoli with increased numbers and enlarged size to boost ribosome biogenesis and translation^37^. Our previous work also revealed that p53 acts as a sensor of nucleolar dysfunction or ribosomal stress in cancer^16,17^. Thus, a thorough elucidation of the molecular basis for p53 response to ribosomal stress would provide molecular insights into the malignant development of cancer cells and offer useful information for future development of anticancer therapies targeting this pathway. In our attempt to address this, we identified SBDS that is encoded by the ribosome biogenesis-associated gene, *SBDS*, as another key player in the ribosomal stress-p53 pathway during tumorigenesis. We found that SBDS is highly expressed in human cancers, and its high level is inversely associated with the survival rate of patients as an unfavorable prognostic factor (Fig. 1). Interestingly, knockdown of SBDS induced ribosomal stress and p53-dependent cell growth arrest (Figs. 2–4). However surprisingly, ectopic expression of this protein also stabilized and activated p53 by untying the MDM2–p53 loop in response to ribosomal stress (Figs. 5, 6), suppressing cancer cell proliferation in vitro (Fig. 7) and tumor growth in vivo (Fig. 8). Therefore, our finding unveils SBDS as a dual regulator of the MDM2–p53 circuit by performing both oncogenic and tumor-suppressive functions in cancer (Fig. 8k).

SDS patients often manifest increased predisposition for malignancies, suggesting the correlation of SBDS mutations with tumorigenesis^3^. Because the incidence of SBDS germline mutation is rare, ~1 in 50,000 births, the role of SBDS mutations in cancer development among a wider population remains largely obscure. As mentioned above, SBDS was highly expressed in a broad range of human cancers and significantly associated with poor prognosis of these cancers (Fig. 1 and S1). These results could be interpreted by the fact that cancer cells need more active ribosome biogenesis to nurture their uncontrolled growth and proliferation. Conversely, knockdown of SBDS induced RPL5 and RPL11 interactions with MDM2, consequently leading to p53 activation (Figs. 2, 3), indicating that SBDS, like other MDM2-binding RPs^16,17^, is required for ribosome homeostasis and its depletion causes ribosomal stress. Interestingly, the results as presented in Fig. 4 and S3 showed that SBDS depletion-induced inhibition of cancer cell proliferation and growth is partially dependent on p53. This is probably because ribosomal stress has also been shown to suppress tumorigenesis through p53-independent pathways, such as c-MYC^38,39^ and TAp73^21,40^. Alternatively, long-term ablation of SBDS may lead to translation impairment^8^ that should constrain cancer cell growth. Therefore, these findings reveal that the endogenous SBDS could play an oncogenic role by exerting its ribosome-associated function and maintaining p53 at a restricted level, and suggest that it could be a biomarker for cancer.

Surprisingly, when SBDS was released from the nucleolus to the nucleoplasm, this protein could act as a tumor suppressor by activating p53 (Fig. 8k). Native SBDS normally resides in the nucleolus and the cytoplasm to function as an essential component involved in rRNA synthesis and ribosome assembly. Ribosomal stress caused by Act. D or 5-FU induced the nuclear localization of SBDS (Fig. 6f), and the nuclear SBDS then bound to the TA domain of p53 (Fig. 6a–d, g) and disrupted the MDM2–p53 association (Fig. 6e), consequently leading to p53 activation. These results also suggest that SBDS might cooperate with RPs in activating p53 in response to ribosomal stress (Fig. 8k), as some RPs, such as RPL11, RPL5, or RPS14, have been shown to activate p53 by interacting with the zinc finger or acidic domain of MDM2^16,17^. Consistent with the above results, ectopically expressed SBDS stabilized p53 by hampering MDM2-mediated p53 ubiquitination-dependent proteolysis (Fig. 5). In agreement with these results, ectopic SBDS suppressed tumor cell growth by inducing p53 activity in vitro and in vivo (Figs. 7, 8). Collectively, although endogenous SBDS is essential for rRNA synthesis in the nucleolus^28^ and for ribosome assembly in the cytoplasm^29^, which may confer its oncogenic activity in cancer (Figs. 1–4), ectopic SBDS in the nucleoplasm plays a tumor-suppressive role in part by activating p53 (Figs. 5–8).

Importantly, our study together with others^17,41^ suggests that malignancies sustaining high expression levels of ribosome-associated genes might be sensitive to the ribosomal stress-inducing agents, such as Act. D and CX-5461. First, a number of ribosome-associated proteins, including SBDS and RPs, can activate the p53 pathway by binding to p53 or MDM2 in response to ribosomal stress (Fig. 8k)^16,17^. Second, these proteins may also exert the antitumor effect independently of p53 by modulating the expression of, for instance, c-Myc^38,39^ and TAp73^21,40^. Furthermore, rapidly growing and proliferating cancer cells need more ribosomes compared with normal somatic cells^17^, also evidenced by the fact that SBDS is often upregulated in cancer and associated with poor prognosis (Fig. 1 and S1). Thus, selective targeting ribosome biogenesis could be a promising strategy for cancer therapy.

Our finding that SBDS binds to the TA domain of p53 suggests that it may also interact with the p53 homologs, TAp63 and TAp73, and display tumor-suppressive activity in p53-negative cancer cells in response to ribosomal stress. Recently, several RPs, including RPL5, RPL11, and RPS14, have been found to associate with TAp73, but not ΔNp73, to overcome MDM2-mediated inactivation, leading to TAp73-induced cancer cell apoptosis^21^. Mechanistically, these RPs directly bind to the TA domain of TAp73 and thus block the association of MDM2 with the same region. It would be interesting to investigate if these ribosome biogenesis-associated proteins, SBDS and RPs, activate p53 homologs in collaboration or independently in cancer cells without functional wild-type p53.

In conclusion, our study as presented here demonstrates that SBDS is not only a potential biomarker or prognostic factor of human cancers, but also could be targeted for cancer therapy, as its knockdown triggers the ribosomal stress-p53 pathway to prevent tumor growth and progression. Also, SBDS could be a crucial activator of p53 and suppress tumor growth in response to the chemotherapeutic treatment, such as Act. D and 5-FU. Hence, our study demonstrates underappreciated dual functions of the SDS-associated protein, SBDS, in cancer development and underscores its potential clinical significance in cancer therapy.

## Supplementary information

Supplementary Information

## References

[1] Boocock GR (2003). Mutations in SBDS are associated with Shwachman-Diamond syndrome. Nat. Genet..

[2] Woloszynek JR (2004). Mutations of the SBDS gene are present in most patients with Shwachman-Diamond syndrome. Blood.

[3] Nakhoul H (2014). Ribosomopathies: mechanisms of disease. Clin. Med. Insights Blood Disord..

[4] Zhang S, Shi M, Hui CC, Rommens JM (2006). Loss of the mouse ortholog of the shwachman-diamond syndrome gene (Sbds) results in early embryonic lethality. Mol. Cell Biol..

[5] Raaijmakers MH (2010). Bone progenitor dysfunction induces myelodysplasia and secondary leukaemia. Nature.

[6] Leung R, Cuddy K, Wang Y, Rommens J, Glogauer M (2011). Sbds is required for Rac2-mediated monocyte migration and signaling downstream of RANK during osteoclastogenesis. Blood.

[7] Tourlakis ME (2012). Deficiency of Sbds in the mouse pancreas leads to features of Shwachman-Diamond syndrome, with loss of zymogen granules. Gastroenterology.

[8] Tourlakis ME (2015). In vivo senescence in the sbds-deficient murine pancreas: cell-type specific consequences of translation insufficiency. PLoS Genet..

[9] Venkatasubramani N, Mayer AN (2008). A zebrafish model for the Shwachman-Diamond syndrome (SDS). Pediatr. Res..

[10] Provost E (2012). Ribosomal biogenesis genes play an essential and p53-independent role in zebrafish pancreas development. Development.

[11] Kastenhuber ER, Lowe SW (2017). Putting p53 in context. Cell.

[12] Levine AJ (2019). The many faces of p53: something for everyone. J. Mol. Cell Biol..

[13] Wade M, Li YC, Wahl GM (2013). MDM2, MDMX and p53 in oncogenesis and cancer therapy. Nat. Rev. Cancer.

[14] Kruse JP, Gu W (2009). Modes of p53 regulation. Cell.

[15] Zhou X, Cao B, Lu H (2017). Negative auto-regulators trap p53 in their web. J. Mol. Cell Biol..

[16] Zhang Y, Lu H (2009). Signaling to p53: ribosomal proteins find their way. Cancer Cell.

[17] Zhou X, Liao WJ, Liao JM, Liao P, Lu H (2015). Ribosomal proteins: functions beyond the ribosome. J. Mol. Cell Biol..

[18] Sanz G, Singh M, Peuget S, Selivanova G (2019). Inhibition of p53 inhibitors: progress, challenges and perspectives. J. Mol. Cell Biol..

[19] Zhou X (2016). Nerve growth factor receptor negates the tumor suppressor p53 as a feedback regulator. eLife.

[20] Zhou X, Hao Q, Liao J, Zhang Q, Lu H (2013). Ribosomal protein S14 unties the MDM2-p53 loop upon ribosomal stress. Oncogene.

[21] Zhou X (2015). Ribosomal proteins L11 and L5 activate TAp73 by overcoming MDM2 inhibition. Cell Death Differ..

[22] Cerami E (2012). The cBio cancer genomics portal: an open platform for exploring multidimensional cancer genomics data. Cancer Discov..

[23] Gao J (2013). Integrative analysis of complex cancer genomics and clinical profiles using the cBioPortal. Sci. Signal..

[24] Lanczky A (2016). miRpower: a web-tool to validate survival-associated miRNAs utilizing expression data from 2178 breast cancer patients. Breast Cancer Res. Treat..

[25] Dai MS (2004). Ribosomal protein L23 activates p53 by inhibiting MDM2 function in response to ribosomal perturbation but not to translation inhibition. Mol. Cell. Biol..

[26] Jin A, Itahana K, O’Keefe K, Zhang Y (2004). Inhibition of HDM2 and activation of p53 by ribosomal protein L23. Mol. Cell. Biol..

[27] Sun XX, Wang YG, Xirodimas DP, Dai MS (2010). Perturbation of 60 S ribosomal biogenesis results in ribosomal protein L5- and L11-dependent p53 activation. J. Biol. Chem..

[28] Ganapathi KA (2007). The human Shwachman-Diamond syndrome protein, SBDS, associates with ribosomal RNA. Blood.

[29] Finch AJ (2011). Uncoupling of GTP hydrolysis from eIF6 release on the ribosome causes Shwachman-Diamond syndrome. Genes Dev..

[30] Karni-Schmidt O (2007). Energy-dependent nucleolar localization of p53 in vitro requires two discrete regions within the p53 carboxyl terminus. Oncogene.

[31] Karni-Schmidt O (2008). p53 is localized to a sub-nucleolar compartment after proteasomal inhibition in an energy-dependent manner. J. Cell Sci..

[32] Dai MS, Lu H (2004). Inhibition of MDM2-mediated p53 ubiquitination and degradation by ribosomal protein L5. J. Biol. Chem..

[33] Macias E (2010). An ARF-independent c-MYC-activated tumor suppression pathway mediated by ribosomal protein-Mdm2 interaction. Cancer Cell.

[34] Bursac S (2012). Mutual protection of ribosomal proteins L5 and L11 from degradation is essential for p53 activation upon ribosomal biogenesis stress. Proc. Natl Acad. Sci. USA.

[35] Chang CJ (2011). p53 regulates epithelial-mesenchymal transition and stem cell properties through modulating miRNAs. Nat. Cell Biol..

[36] Oliner JD (1993). Oncoprotein MDM2 conceals the activation domain of tumour suppressor p53. Nature.

[37] White RJ (2005). RNA polymerases I and III, growth control and cancer. Nat. Rev. Mol. Cell Biol..

[38] Zhou X, Hao Q, Liao JM, Liao P, Lu H (2013). Ribosomal protein S14 negatively regulates c-Myc activity. J. Biol. Chem..

[39] Dai MS, Arnold H, Sun XX, Sears R, Lu H (2007). Inhibition of c-Myc activity by ribosomal protein L11. EMBO J..

[40] Zhang M, Zhang J, Yan W, Chen X (2016). p73 expression is regulated by ribosomal protein RPL26 through mRNA translation and protein stability. Oncotarget.

[41] Bywater MJ (2012). Inhibition of RNA polymerase I as a therapeutic strategy to promote cancer-specific activation of p53. Cancer Cell.

