# Supplementary material for: Dual regulation of p53 by the ribosome maturation factor SBDS
Source: Cell Death Dis. 2020 Mar 20;11(3):197. doi: 10.1038/s41419-020-2393-4 (PMC7083877; doi:10.1038/s41419-020-2393-4)

Supplementary Information for

Dual regulation of p53 by the ribosome maturation factor SBDS

Qian Hao[1,2,*], Jieqiong Wang[1,2,3], Yajie Chen[1,2], Shanshan Wang[1,2], Mingming Cao[1,2], Hua Lu[4], and Xiang Zhou[1,2,5,6,*]

[1] Fudan University Shanghai Cancer Center and Institutes of Biomedical Sciences, Fudan University, Shanghai 200032, China;

[2] Department of Oncology, Shanghai Medical College, Fudan University, Shanghai 200032, China;

[3] Present address: Department of Biochemistry & Molecular Biology, Tulane University School of Medicine, New Orleans, LA 70112, USA;

[4] Department of Biochemistry & Molecular Biology and Tulane Cancer Center, Tulane University School of Medicine, New Orleans, LA 70112, USA;

[5] Key Laboratory of Medical Epigenetics and Metabolism, Fudan University, Shanghai 200032, P. R. China;

[6] Key Laboratory of Breast Cancer in Shanghai, Fudan University Shanghai Cancer Center, Fudan University, Shanghai 200032, China.

Running Title: Two sides of SBDS in cancer

[*] Corresponding Authors:

Xiang Zhou, Fudan University Shanghai Cancer Center and Institutes of Biomedical Sciences, Fudan University, Shanghai 200032, P. R. China. Email: xiangzhou@fudan.edu.cn

Qian Hao, Fudan university Shanghai Cancer Center, Fudan University, Shanghai 200032, P. R. China. Email: qhao15@hotmail.com

This PDF file includes:

Supplementary Figure legends

Supplementary Figures S1 to S6

Supplementary Figure Legends

Figure S1 SBDS is overexpressed in human cancers. (A) SBDS is upregulated in cancer versus normal tissues, including renal cell carcinoma, lung carcinoma, leukemia, myeloma, lymphoma, cervical carcinoma, and tongue and oral cavity carcinomas. (B) SBDS is inclined to be amplified or overexpressed in most human cancers.

Figure S2 RNAi-mediated knockdown of SBDS in HCT116$^{p53+/+}$ and HCT116$^{p53-/-}$ cell lines. (A) Knockdown of SBDS by siRNA. HCT116$^{p53+/+}$ and HCT116$^{p53-/-}$ cells were transfected with SBDS siRNA or control, and harvested at day 1, 2, 3, and 4 post transfection for IB analysis using antibodies as indicated. (B) Knockdown of SBDS by shRNAs. HCT116$^{p53+/+}$ and HCT116$^{p53-/-}$ cells were infected with lentiviruses containing SBDS shRNAs or control, and harvested at day 2, 3, 4, and 5 post infection for IB analysis using antibodies as indicated.

Figure S3 Knockdown of SBDS inhibits cancer cell colony-forming ability. HCT116$^{p53+/+}$ (A) and HCT116$^{p53-/-}$ (B) cells were transfected with SBDS siRNA followed by the colony formation assay. Quantification of colonies is shown in the right panels.

Figure S4 Ectopic SBDS leads to p53 accumulation in the nucleus and the nucleolus. H460 cells were transfected with pEnter or Flag-SBDS, and analyzed by IF staining. The white arrows indicate the nucleoli expressing the nucleolar marker, Fibrillarin.

Figure S5 SBDS slightly binds to MDM2. H1299 cells were transfected with combinations of plasmids as indicated. The co-IP was conducted using the anti-Flag antibody and the bound complexes were detected by IB using antibodies as indicated.

Figure S6 MDM2 binds to p53 in the nucleus in response to ribosomal stress. Cells were treated with or without Act. D followed by a proximity ligation assay using antibodies as indicated.

Figure S1

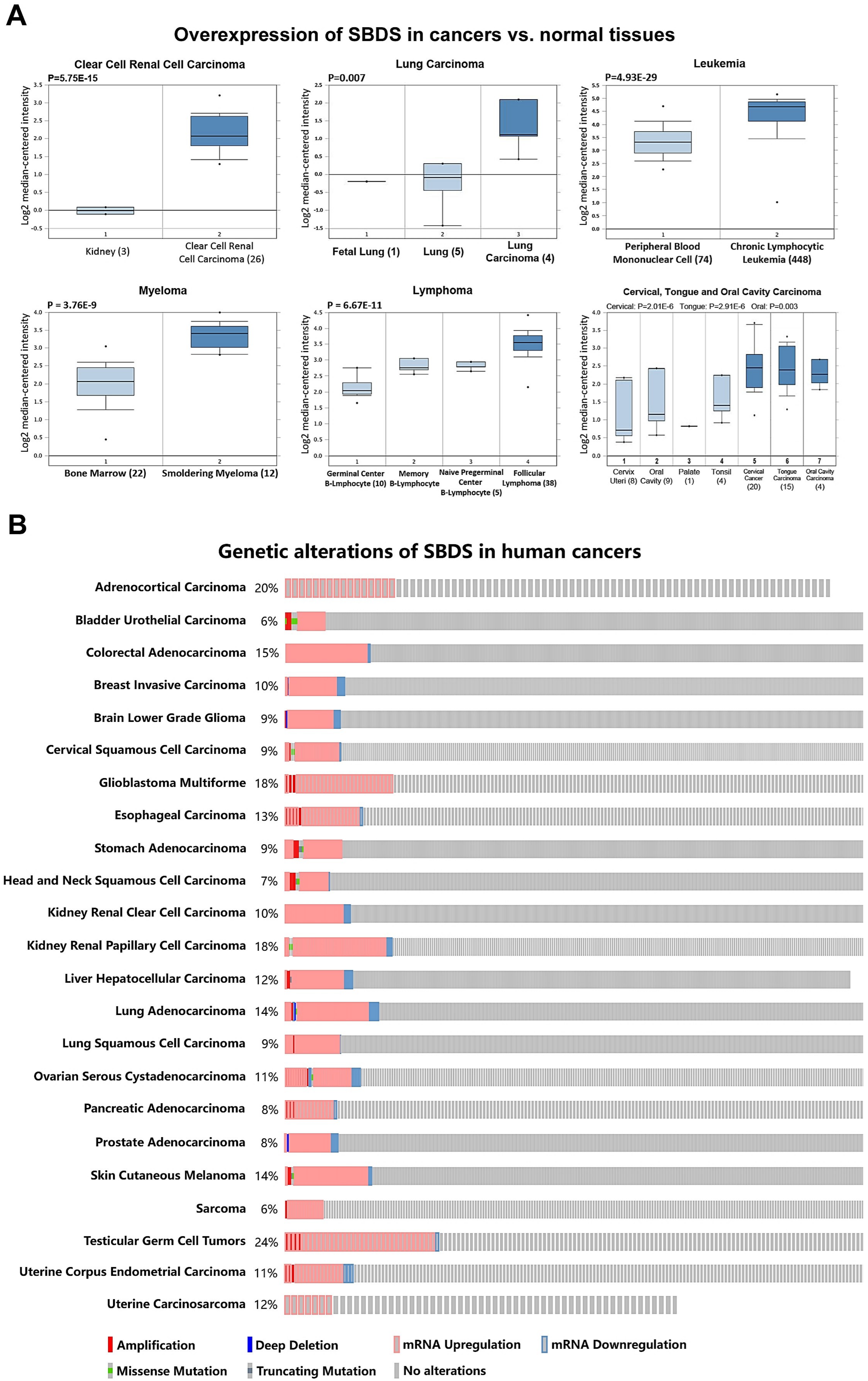

A
Overexpression of SBDS in cancers vs. normal tissues
Clear Cell Renal Cell Carcinoma
P=5.75E-15
Kidney (3)
Clear Cell Renal Cell Carcinoma (26)
Lung Carcinoma
P=0.007
Fetal Lung (1)
Lung (5)
Lung Carcinoma (4)
Leukemia
P=4.93E-29
Peripheral Blood Mononuclear Cell (74)
Chronic Lymphocytic Leukemia (448)
Myeloma
P = 3.76E-9
Bone Marrow (22)
Smoldering Myeloma (12)
Lymphoma
P = 6.67E-11
Germinal Center B-Lmphocyte (10)
Memory B-Lymphocyte
Naive Pregerminal Center B-Lymphocyte (5)
Follicular Lymphoma (38)
Cervical, Tongue and Oral Cavity Carcinoma
Cervical: P=2.01E-6 Tongue: P=2.91E-6 Oral: P=0.003
Cervix Uteri (8)
Oral Cavity (9)
Palate (1)
Tonsil (4)
Cervical Cancer (20)
Tongue Carcinoma (15)
Oral Cavity Carcinoma (4)
Log2 median-centered intensity
B
Genetic alterations of SBDS in human cancers
Adrenocortical Carcinoma 20%
Bladder Urothelial Carcinoma 6%
Colorectal Adenocarcinoma 15%
Breast Invasive Carcinoma 10%
Brain Lower Grade Glioma 9%
Cervical Squamous Cell Carcinoma 9%
Glioblastoma Multiforme 18%
Esophageal Carcinoma 13%
Stomach Adenocarcinoma 9%
Head and Neck Squamous Cell Carcinoma 7%
Kidney Renal Clear Cell Carcinoma 10%
Kidney Renal Papillary Cell Carcinoma 18%
Liver Hepatocellular Carcinoma 12%
Lung Adenocarcinoma 14%
Lung Squamous Cell Carcinoma 9%
Ovarian Serous Cystadenocarcinoma 11%
Pancreatic Adenocarcinoma 8%
Prostate Adenocarcinoma 8%
Skin Cutaneous Melanoma 14%
Sarcoma 6%
Testicular Germ Cell Tumors 24%
Uterine Corpus Endometrial Carcinoma 11%
Uterine Carcinosarcoma 12%
Amplification
Deep Deletion
mRNA Upregulation
mRNA Downregulation
Missense Mutation
Truncating Mutation
No alterations

Fgiure S2

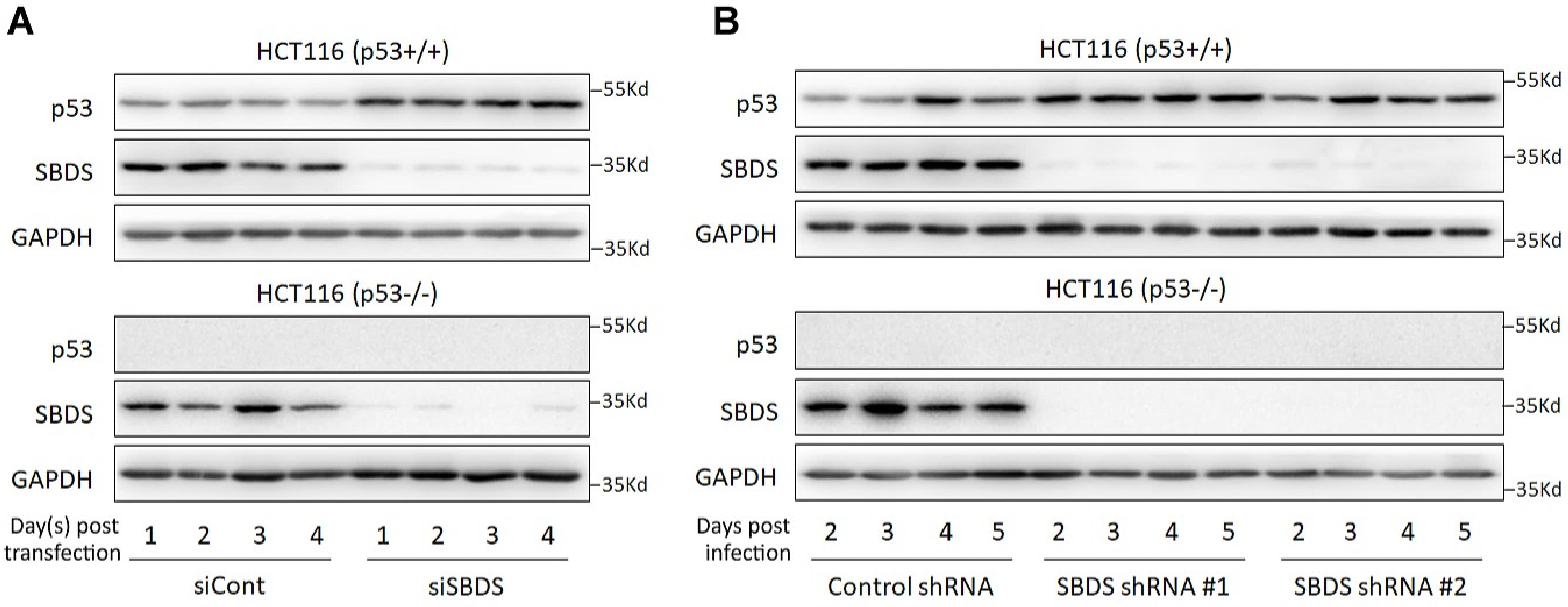
A
HCT116 (p53+/+)
p53
SBDS
GAPDH
55Kd
35Kd
35Kd
HCT116 (p53-/-)
p53
SBDS
GAPDH
55Kd
35Kd
35Kd
Day(s) post transfection
1 2 3 4 1 2 3 4
siCont
siSBDS
B
HCT116 (p53+/+)
p53
SBDS
GAPDH
55Kd
35Kd
35Kd
HCT116 (p53-/-)
p53
SBDS
GAPDH
55Kd
35Kd
35Kd
Days post infection
2 3 4 5 2 3 4 5 2 3 4 5
Control shRNA
SBDS shRNA #1
SBDS shRNA #2

Figure S3

A

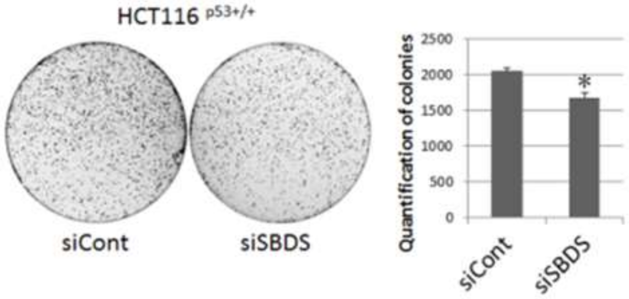
HCT116 p53+/+
siCont
siSBDS
Quantification of colonies
2500
2000
1500
1000
500
0
*
siCont
siSBDS

B

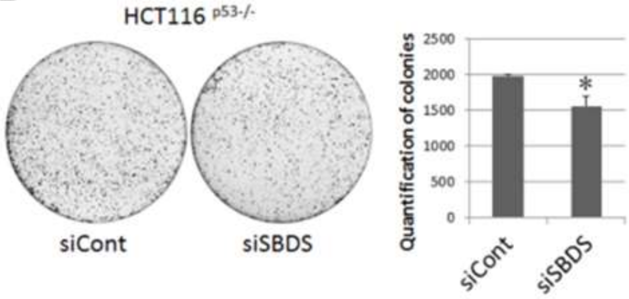
HCT116 p53-/-
siCont
siSBDS
Quantification of colonies
2500
2000
1500
1000
500
0
*
siCont
siSBDS

Figure S4

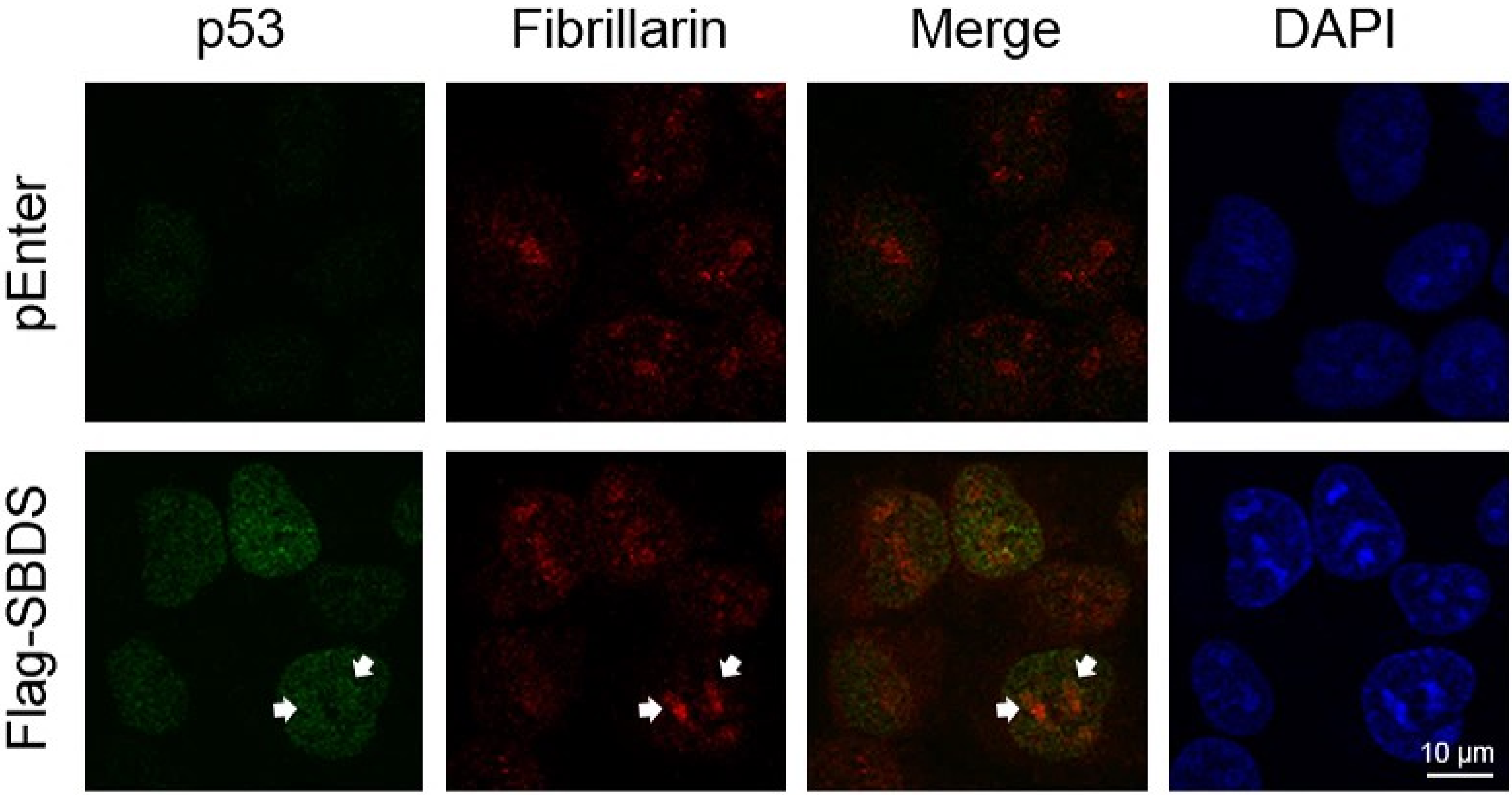

Figure S5

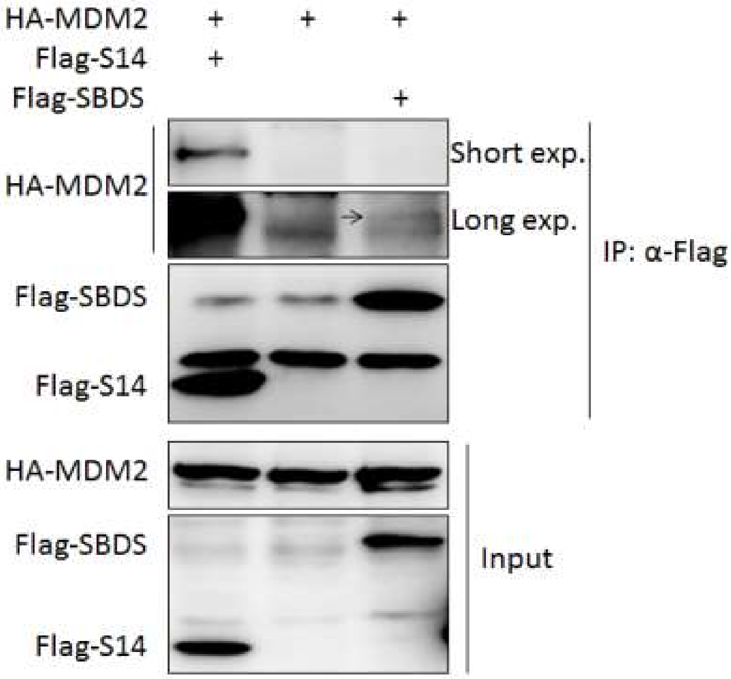
HA-MDM2 + + +
Flag-S14 +
Flag-SBDS +
HA-MDM2
Short exp.
Long exp.
IP: α-Flag
Flag-SBDS
Flag-S14
HA-MDM2
Flag-SBDS
Input
Flag-S14

Figure S6

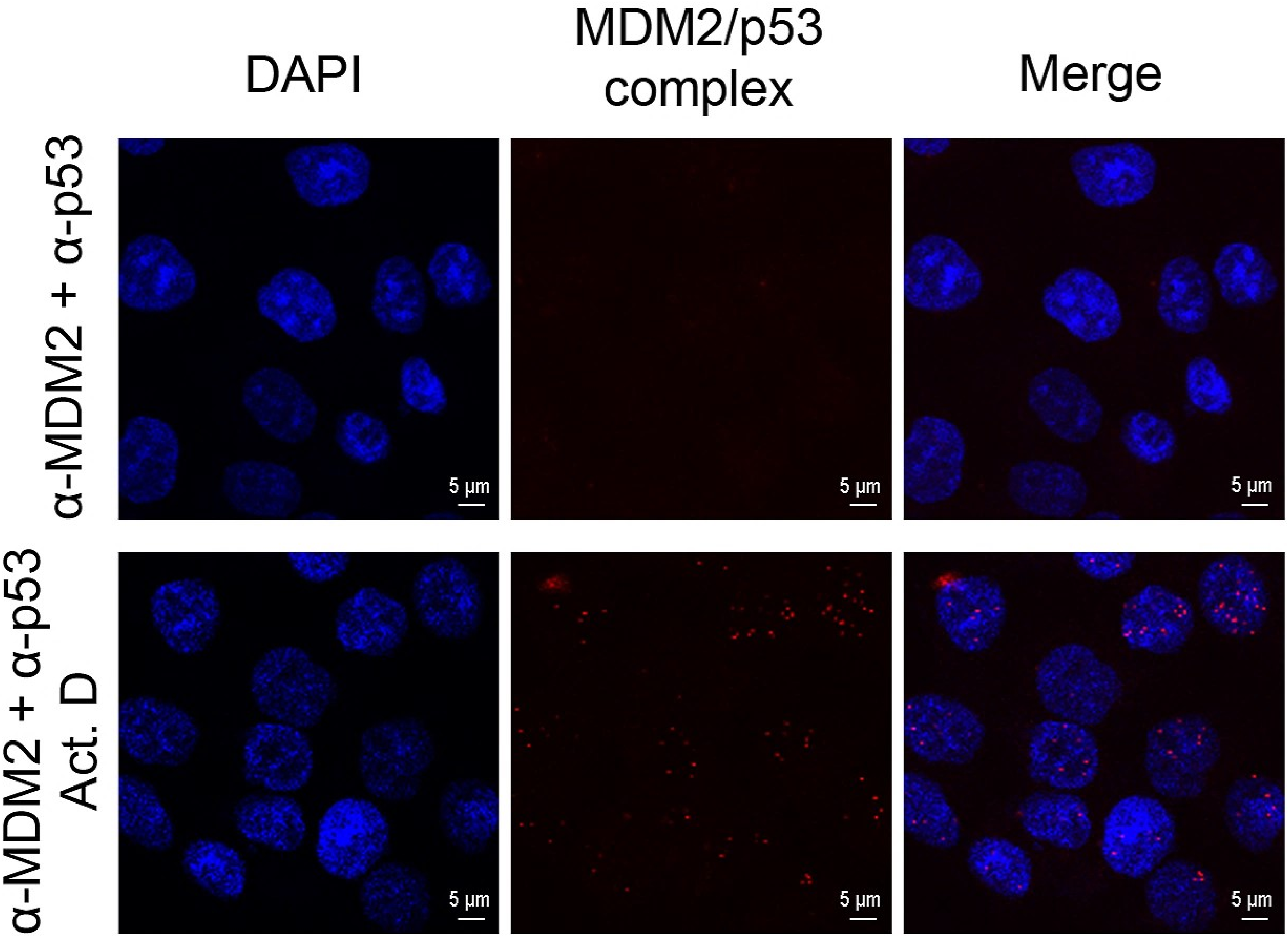

Supplement: Supplementary file 1 — Supplementary Information [file 41419_2020_2393_MOESM1_ESM.pdf]
